# The use of paracetamol for first-line treatment of acute sore throat. A descriptive generic qualitative study of GPs and patients

**DOI:** 10.1080/13814788.2021.1912730

**Published:** 2021-05-05

**Authors:** Kimberley De Vocht, Tycho Debie, Hilde Bastiaens, Sibyl Anthierens

**Affiliations:** Department of Family Medicine and Population Health, University of Antwerp, Antwerp, Belgium

**Keywords:** Acute sore throat, paracetamol, patients, general practitioners, qualitative research

## Abstract

**Background:**

Paracetamol is recommended as first-line treatment for an acute sore throat. However, in primary care, antibiotics are still frequently prescribed as first-line management for sore throat.

**Objectives:**

We aimed to explore the views and experiences of general practitioners (GPs) and patients regarding paracetamol for sore throat to understand why guidelines are often not adhered to.

**Methods:**

A descriptive qualitative study with semi-structured interviews was conducted with a purposive sample of eight GPs and nine patients in Antwerp (Belgium). Data was analysed using thematic content analysis.

**Results:**

The mean age of GPs was 42.4 years and of patients 51.4 years. Patients want reassurance and pain relief. Many patients do not self-manage their acute sore throat with paracetamol before consulting their GP. GPs often wrongly assume that the patient has already used pain relief without actually exploring this. Patients who do use paracetamol, do not know how to use it effectively. This leads to the perception and beliefs that it is insufficient to treat acute sore throat and when prescribed will lead to dissatisfaction. Patients generally accept the GP’s recommended treatment when given a thorough explanation, since they trust their GP’s expertise.

**Conclusion:**

GPs play a major role in educating patients about paracetamol as effective pain-relieving treatment in acute sore throat. By actively exploring the patients’ ideas, concerns and expectations (ICE), patients’ satisfaction and guideline adherence could be improved.

KEY MESSAGES

- GPs often wrongly assume patients’ expectations and overestimate their knowledge about paracetamol.
- Both GPs and patients perceive paracetamol as a weak painkiller for acute sore throat; for patients this can be attributed to the incorrect use.
- Patients’ primary source of information about medication is their GP, whom they deeply trust

## Introduction

In Belgium, having an acute sore throat is a common reason to consult the general practitioner (GP) [1]. The recommended approach in Flemish guidelines to relieve pain caused by a sore throat is using a time-based approach of paracetamol, 1 gram four times per day. However, patients’ knowledge about the use of paracetamol appears to be substantially insufficient or incorrect [2]. A non-steroidal anti-inflammatory drug (NSAID) is recommended as an alternative, keeping the side-effects, co-morbidities and possible drug interactions in mind. Antibiotics are only recommended for patients who are severely ill, at risk for severe complications, or when a peritonsillar abscess is suspected [3–5]. Many campaigns and studies target the overuse of antibiotics, looking into the perceptions and practices of their use by both clinicians and patients [6].

Worldwide, most guidelines focus on antibiotics as a treatment [7]. Only 11 guidelines, like the European Society for Clinical Microbiology and Infectious Diseases, Germany, Sweden and the UK, explicitly advise the combination of paracetamol and a NSAID [7–10]. In contrast, the Dutch guideline discourages the use of NSAIDs [3].

Guidelines provide tools to explain to patients why antibiotics are not required for an acute sore throat and advise to correlate their explanation to the ICE of the patient [3–5]. ICE is an acronym and refers to a communication technique to elicit information about patients’ ideas, concerns and expectations [11–13].

This study aims to explore the views on and experiences with using paracetamol in sore throat in depth, from the perspective of both patients and GPs. Bringing these views and experiences together can provide a better understanding of GPs’ decision making and patients’ expectations. This may help to identify strategies to improve the adherence to recommended treatment for acute sore throat.

## Methods

### Study design

Since we aimed to explore the experiences and perceptions of GPs and patients concerning paracetamol as first-line treatment for an acute sore throat, a descriptive generic qualitative study was carried out [14–17].

### Selection of participants

Purposeful sampling was used, aiming to achieve maximal variation in age, gender, type of practice (GPs) and occupation (patient) and provide a diversified pool of perspectives [18,19].

General practitioners working at least half-time at urban and rural locations in Antwerp were eligible for participation. GPs in training were excluded from the study. Based on these criteria, 16 GPs were contacted by telephone or e-mail. Eight GPs opted to participate.

Patients older than 18 years, who attended their GP practice for an acute sore throat in the last 12 months, were eligible. Patients with physical or mental disabilities or those deemed to be at risk were excluded from study participation. To recruit patients, project information sheets were distributed at different locations, including retirement homes, GP waiting rooms and youth service centres. Of the 21 patients who were interested in participating, nine fulfilled the eligibility criteria.

A diverse group of eight GPs and nine patients were interviewed (Table 1). The mean age of GPs was 42.4 years (SD 13.4) and of patients 51.4 years (SD 25.7). Five patients consulted their GP one to two months earlier for a sore throat, the other four between six to nine months. Interviews with patients were conducted between December 2016 and August 2017 and lasted between 13 and 33 min, with an average of 25 min. Interviews with general practitioners were conducted between December 2016 and April 2018 and lasted between 33 and 69 min, with an average of 45 min.

**Table 1.** Participant Characteristics.

Number	Gender	Age (years)	Setting (GP)/Occupation (patient)
General practitioners			
1	♂	52	Solo practice
2	♀	46	Group practice
3	♂	63	Solo practice
4	♀	27	Group practice
5	♀	29	Group practice
6	♂	28	Group practice
7	♀	36	Practice with two GPs
8	♀	58	Group practice
Patients			
1	♀	20	Socio-Educational Care Work Student, single
2	♀	55	Housewife, married
3	♀	42	Civil Servant, married
4	♂	23	Logistician, single
5	♂	50	Sales Director, remarried
6	♂	82	Retired Car Mechanic, married
7	♂	71	Retired History Professor, nursing home resident, widower
8	♀	96	Former housewife, nursing home resident, widow
9	♂	24	Biology Student, single

The scope of our study was pragmatic and explorative. For pragmatic reasons, we were limited in the number of interviews that could be undertaken; however, two key considerations for the sampling methods were taken into account, appropriateness and adequacy to sufficiently answer the research question [20,21]. For patient interviews, no new information was found after analysing six interviews. For GPs, data is sufficient, meaning enough in-depth data to show variety of the phenomenon is available.

### Data collection

Semi-structured interviews were chosen as data collection method to allow in-depth analysis of individual perspectives [15]. Separate interview guides for GPs and patients were developed, based on the existing information on exploring the ICE of the patient during the consultation [11–13,15]. The interview guides were piloted and refined.

Patient interviews focussed on experiences with consulting a GP about an acute sore throat and assessed their views on medication for pharyngitis with explicit focus on paracetamol. GP interview topics explored views on consultations for acute sore throat and its treatment, before probing deeper into the use of paracetamol. A short overview of the topics is provided in Table 2.

**Table 2.** Summarised translation of the topic guides for GPs and patients.

GPs were asked to think about a recent consultation with patients with acute sore throat	Flow of an average consultation for sore throat Management of sore throat. Information provision to patients. Pain relief medication for acute sore throat. Role of guidelines, consultation time and colleagues in the management of sore throat. Concrete experiences with providing pain relief medication and paracetamol to patients.
Patients were asked to think about a consultation they had with their GP for acute sore throat	Exploring own management of acute sore throat and reasons to consult GP. General experiences of a consultation for acute sore throat with GP. Experience with and knowledge of paracetamol. Experiences with and views on use of paracetamol for acute sore throat.

The interviews were conducted face to face by two researchers (TD interviewed GPs and KDV interviewed patients), who were medical students undertaking the study as part of their Master’s thesis. The interviews were audio-recorded and transcribed verbatim. Afterwards, the audio material was destroyed and transcriptions securely saved for 20 years by the University of Antwerp.

### Analysis

The data was coded inductively through thematic content-analysis, using the following steps. Transcripts were read and meaningful fragments and relevant quotes were selected and coded. Coding was first open with possible reservations in a memo. Next, codes with similar meaning were grouped and bundled in major themes [19,22].

Peer debriefing, a reflexive attitude of our impact as a researcher on the data and analysis, complementary data (GPs and patients) and researcher triangulation were used to increase our study’s credibility. One researcher (TD) focussed on the interviews with GPs and the other one (KDV) on those with the patients. Two interviews were coded independently by all authors, critically evaluated for interview techniques and afterwards discussed extensively. Another patient interview was double coded. Further, through discussion between the researchers and supervisors at different stages in the analytical process, the personal interpretation of the data could be broadened and balanced. Finally, as a last step, the results from GPs and patients were integrated with each other.

### Ethics

The Ethics Committee of the University of Antwerp approved this study (16/26/275) on 10/10/2016. Written informed consent was obtained before each interview.

## Results

### Theme description

Three main themes influencing the use of paracetamol as a first-line treatment of acute sore throat in GPs and patients were found (see Table 3). They will be discussed one by one below.

**Table 3.** Overview of the themes for GPs and patients.

Themes for GPs	Themes for patients
Assumptions about patients’ expectations, self-care activities and paracetamol use The role of clinical symptoms and the place of medication in management of acute sore throat Adherence to guidelines and influence of peers	Reasons and expectations for consulting the GP with an acute sore throat. Patients’ knowledge and use of paracetamol. Perceived severity of the acute sore throat and role of previous treatment experiences.

#### Themes for GPs

##### Assumptions about patients’ expectations, self-care activities and paracetamol use

Several GPs indicated that patients wanted immediate treatment and pain relief, so they felt more inclined to prescribe a ‘kill or cure remedy’.

*There are a lot of patients who say, ‘Okay doctor, I'm going to do that’’, but you also have answers like:*

*“I’ve tried that, that's not strong enough for me. You don’t take me seriously, because you only prescribe paracetamol”. (…) Then they’re saying, ‘paracetamol is really just some kind of candy to me”, I need the real deal. (GP4)*

A couple of GPs systematically explored the patients’ expectations, which helped them better understand the patient. Therefore, the discussion on the treatment went more easily and resulted in a satisfied patient. Others never explored the ICE in their consultations.

*I've never asked a patient “what do you expect from the treatment?”, but I cannot imagine that a patient would consult me with a sore throat and doesn’t expect to be helped quickly. (GP3)*

Some GPs automatically assume that patients have already started taking paracetamol for an acute sore throat or were advised to do so by their pharmacist. For these GPs, it does not seem appropriate to propose this well-known self-treatment readily available over the counter (OTC).

However, other GPs pointed out that patients used paracetamol in a lower dosage (e.g. 500 mg) than the recommended treatment dose and did so according to pain levels (one to two times per day). Consequently, these patients tend to perceive it as not working sufficiently. Certain GPs took the time to educate their patients about the correct use of paracetamol and reported patients were satisfied with the explanation. In congtrast, other GPs seem to not routinely discuss this due to, among others, time constraints.

Several practitioners experienced a cultural push of taking medication in Belgium. They feel that many patients take pills too quickly for minor complaints and feel pressured to prescribe medication when they are consulted. Furthermore, some GPs feel that suggesting patients to purchase an OTC medication is not a real prescription nor meeting patients’ expectations.

Overall, participating GPs seemed to assume that patients want to be helped as quickly as possible, despite not exploring their expectations.

##### The role of clinical symptoms and the place of medication in management of acute sore throat

Some GPs prefer to base their treatment decision on a distinction between a viral and bacterial cause, others prescribe according to the severity of the patients’ symptoms. When an antibiotic was given, the focus seemed to shift from symptomatic pain control to only causal treatment.

*So, if you see a purulent throat, then I think you can decide on a clinical basis that there’s a bacterial infection going on. If you see a really red throat, chances are real that there is a streptococcus involved. In those cases, you give antibiotics. (GP1)*

Several participants indicate to choose paracetamol because of its adequate analgesic effect and few side-effects in contrast to NSAIDs. Others prefer a NSAID because of a perceived stronger analgesic effect.

*As ibuprofen has or may have a lot of side-effects, I try to avoid this product. (…) Anything that can be treated with only paracetamol, has my preference’. (GP5)*

To summarise, GPs’ views on medical treatment, their goals and the task they see for themselves, are important elements that influence whether paracetamol will be recommended as a first-line treatment for sore throat.

##### Adherence to guidelines and influence of peers

Several GPs indicated that they adhere firmly to guidelines. Others stated that they are not familiar with the guidelines, because they feel that guidelines should be updated more regularly or do not apply to their practice situation. In addition, some GPs pointed out that there is no straightforward policy about managing an acute sore throat, therefore many different approaches and products can be recommended.

*So, some colleagues base their management on their experience, instead of evidence. Usually, they are the slightly older ones, like in the solo practice where I was trained: Yeah, I’ve experienced that already if I do this or that… You must do it like this. Gee, guidelines… they shouldn't tell me what to do. I know that myself. (GP5)*

In the interviews, competition between practitioners was cited as a factor limiting the adherence to scientific guidelines. The idea that fellow GPs might prescribe antibiotics, resulting in them being perceived as ‘the better doctor’, seems to make it more difficult for some GPs to stay true to their evidence-based principles.

*Colleagues easily write antibiotics and do not change their policies when they are told in supplementary training. I think it’s a pity for two reasons. First, because I think that as a doctor you also have a responsibility to manage antibiotics responsibly. And second, to base your care on certain evidence, because (…) when they visit me with a sore throat without getting antibiotics and they go to a colleague two days later and they do get antibiotics immediately without a fuss. Who is the bad doctor in the eyes of the patient, do you think? (GP6)*

In summary, GPs differ in their views and value given to guidelines as a tool to direct evidence-based management. In addition, close colleagues also influence the prescribing practice of the GP.

#### Themes for patients

##### Reasons and expectations for consulting the GP for an acute sore throat

Patients chose to book a GP consultation for acute sore throat for the following reasons: when the pain was too intense, if the pain had persisted for a couple of days, or if it was associated with fever or coughing.

When consulting the GP for an acute sore throat, patients mainly wanted pain relief, next to a clinical examination to investigate the cause, and a prognosis. Only a few patients expected certification for time off work. Often patients wanted a medication prescription from their GP without explicitly stating which drug, as long as it would treat or accelerate the healing of their sore throat.

*I expect the GP to prescribe me something that will relieve or diminish the pain the next day. I think that there are problems with the excessive use of antibiotics, so if I can heal with something that doesn’t contain it, I am favouring that. (P7)*

If the GP explained that the cause was most likely a virus and that an antibiotic could not treat this, patients were more likely to comprehend the recommendation for paracetamol. Most patients trusted the GP’s expertise and would therefore rarely doubt their advice.

I never questioned my GP. I assume he has studied very hard; he is a very skilled man. I know I can't do it better myself. He has been able to treat a lot of people, like my family. So, I trust his opinion. (P9)

##### Patients’ knowledge and use of paracetamol

When patients were asked what they thought paracetamol was used to treat, most mentioned headache and very few could name any other indications for its use. This would suggest that patients are not aware that paracetamol is an effective treatment for acute sore throat.

*I think I only use paracetamol when I’m having a headache due to fever… In my head is just like: paracetamol is for a headache. (P1)*

When questioned about paracetamol’s side-effects, they believed it could cause addiction, anticoagulation, kidney damage and could be lethal. Only one patient named the hepatotoxic effect as a possible side-effect. Therefore, patients did not want to take too high a dose, nor take paracetamol too often and only once or twice a day when the pain was unbearable. Sometimes patients experienced NSAIDs to be more effective for pain relief than paracetamol.

*I know that if you take too much paracetamol, you’ll die, that your blood is too diluted. If you take two 500 milligrams after each other, you could faint. (P4)*

To summarise, in our study patients’ knowledge of paracetamol seems very limited. As patients sometimes fear side-effects, they experiment with taking paracetamol in less frequent and lower doses than recommended in a pain-based rather than a time-based manner.

##### Perceived severity of the acute sore throat and role of previous treatment experiences

In general, patients described an acute sore throat as a mild condition, which your immune system should be able to handle. Most participants had already started taking lozenges, syrups or sprays at home before consulting the GP. Only a few patients stated that they had started with paracetamol before visiting the GP. At the same time, other patients wanted to avoid taking drugs in general.

The treatment of an earlier episode was an important factor determining patients’ expectations when having a new episode of acute sore throat. If paracetamol had been advised during a previous consultation, patients were inclined to use this again. If the GP told them previously that they had a severe throat infection requiring antibiotics, patients assumed that antibiotics were always necessary for a similar presentation.

*I had really thick, swollen, white dots in my throat, so that wasn’t really bearable. The GP looked at my throat and said, “This clearly needs antibiotics”, so I thought it might be better because the pain was indeed worse (…) I've never actually tried paracetamol for a sore throat myself really. (P1)*

##### Comparison of GPs’ and patients’ results

Our study found that patients with acute sore throat often attend their GP for reassurance or (quick) pain relief, without explicitly mentioning medication type. Because prescribing OTC products is not seen as a ‘medical act’, GPs may think they were consulted and paid without offering their patients a beneficial solution.

Furthermore, it appears that GPs do not actively explore if patients have already self-commenced an OTC painkiller prior to their consultation, butsome patients had indeed already initiated a therapy at home to reduce their symptoms. This was usually guided by previous advice from a GP for a similar episode of acute sore throat.

Both patients and several GPs perceive paracetamol as a ‘weak’ painkiller. The aforementioned GPs think patients will feel like they are not seriously regarded if they suggest paracetamol as the only treatment. Therefore, they do not advise it as a first-line treatment, although our patient interviews do not support this. Patients indicate to follow what their GP recommends and tend to put great trust in their GP’s professional judgement.

For multiple patients, their GP was the primary source of medication information but they describe that the use of paracetamol is not commonly explained to them. Therefore, many patients do not use paracetamol correctly. These patient findings contrast the ideas of the GPs who assume that patients are familiar with the use of paracetamol as a treatment for acute sore throat.

## Discussion

### Main findings

This study explored the views and experiences of GPs and patients with the use of paracetamol as first-line treatment for an acute sore throat. Comparing these perspectives resulted in interesting differences.

For GPs, a complex interplay of the three themes influenced why practice deviated from guidelines. First, the assumed patients’ expectations mentioned by several GPs, seem to differ remarkably from what patients expect. GPs want to respond to patients' expectations but do not explicitly ask them. Therefore, their management changes due to new assumptions. Second, some GPs perceived paracetamol as too weak and were consequently unlikely to recommend it. Third, as several GPs consider guidelines to be inconsistent or do not actively use them, their knowledge is mainly based on their own experiences and ideas. Moreover, those GPs are influencing the adherence to the guidelines of surrounding GPs.

Patients are mainly expecting pain relief and often expect a medication prescription. However, they did not instinctively think of paracetamol as a possible treatment for an acute sore throat. The ones who already experienced using paracetamol for acute sore throat often misused it (dosage and duration) and, therefore, assumed it was not effective in alleviating their symptoms. Overall, patients in this study were very open to the professional opinion of their GPs and would be satisfied with a paracetamol prescription when given a proper explanation.

### Comparison with existing literature

Patients who consulted a GP for an acute sore throat, mainly expected pain relief, a clinical examination and information about the prognosis [23–25]. Research shows that, in general, patients’ ICE is rarely questioned by the GP during a consultation for sore throat [11]. Both GPs and patients in our study stated to rarely discuss the patients’ ICE, resulting in a blind spot in the consultation. This finding is in contrast to a study conducted in Ghent in 2009, where most GPs assessed some or all ICE components [12].

When the GP explicitly questioned the patient’s ICE, it was observed that medication was less frequently prescribed [12]. In respiratory tract infections (RTIs), antibiotics are frequently prescribed to meet the patients’ expectations [26]. Worldwide, the explicit request for an antibiotic prescription for an RTI is declining [27]. Hence, educational messages explaining the viral cause of RTIs and the limited use of antibiotics in viral infections, are used as a main strategy to counter those expectations [26,28]. According to our participants, such a message could easily reassure them and help them to understand the final management.

In the shared decision-making model, the GP can involve the patient to clarify his ideas, providing an opportunity to correct possible inaccuracies. It seems important to adjust this explanation to the patients’ ICE and self-commenced treatment [13]. This is consistent with our study, where GPs who addressed the patients’ expectations, experienced a more efficient discussion resulting in high patient satisfaction.

When the communication skills of GPs were encouraged with interactive workshops or during internet training, this resulted in fewer antibiotic prescriptions for RTIs [29,30]. Furthermore, a patient-oriented booklet about the (non-)antibiotic management of RTIs, supported GPs in informing patients [30]. Factors limiting using such communication techniques were time constraints, diagnostic uncertainty and scarce availability of patient-centred information leaflets [26]. Some of those factors were also expressed by GPs during the interviews.

### Implications for clinical practice

First, it is vital that GPs actively explore the patient’s ICE to personalise the treatment and stimulate compliance. Furthermore, GPs should explore which specific treatment the patient has already started since this can range from nothing to taking a plethora of medications. This will allow the doctor to understand better the patient’s level of understanding about acute sore throat treatment, and decipher the best treatment and advice for the patient. In conclusion, exploring the patients’ ICE is one apparent factor that could improve adherence to the guidelines.

Secondly, based on the finding that most patients do not recognise paracetamol as an effective treatment for a sore throat, it is important that GPs present it as a first-line treatment. Patients should be thoroughly informed about the dose and frequency of paracetamol for an optimal effect because it was noted that they often do not take it correctly. The GP can play a major role in altering the patient perception of paracetamol as a pain-relieving treatment through education.

Further, practising the principles of shared decision-making in sore throat consultations could be an important improvement in compliance and patients’ satisfaction. GPs could discuss the management options with their patients based on the existing guidelines or refer to evidence-based and patient-oriented websites or leaflets [13,26].

Finally, GPs in our study seem to value their peers’ opinions, to such an extent that some GPs felt compelled to deviate from evidence-based principles. Therefore, it seems reasonable to stimulate discussion of the management of common pathologies on a local level to improve general guideline adherence.

### Strengths and limitations

The interviewers had limited experience with qualitative research, which was compensated by the supervisors’ experience and cooperation. The researchers also aimed to keep the credibility as high as possible by peer-debriefing, a reflexive attitude and triangulation between researchers and supervisors.

The inclusion criteria that allowed patients to be involved up to 12 months after their consultation for acute sore throat may have been detrimental to their ability to recall events. However, more than half of the patients consulted their GP one to two months before the interview and the other half no more than nine months without significant differences in their views and experiences.

All participants had a Western cultural background and we did not reach people in vulnerable situations. We do, however, feel that we managed to gather enough relevant insights to meet the explorative study aims but we cannot draw conclusions on the perception of patients from different cultural backgrounds. This should be investigated further.

We consider it an advantage to combine both GPs’ and patients’ views about this topic. In this way, it was possible to highlight the main differences between the two groups, which adds a new and comprehensive perspective.

## Conclusion

In this qualitative research paper, we brought together views and experiences of GPs and patients on paracetamol as a first-line treatment of acute sore throat. It showed that GPs often assume their patients’ ICE without actively exploring them. By actively exploring the patients’ ICE, patients’ satisfaction and guideline adherence could be improved. In contrast to GPs’ assumption of paracetamol as a well-known product, patients lack knowledge about paracetamol for acute sore throat and its correct use. Therefore, GPs play a major role in educating patients about paracetamol, especially since patients generally trust their expertise.
